# Viruses Teaching Immunology: Role of LCMV Model and Human Viral Infections in Immunological Discoveries

**DOI:** 10.3390/v11020106

**Published:** 2019-01-27

**Authors:** Mohamed S. Abdel-Hakeem

**Affiliations:** 1 Penn Institute for Immunology, Perelman School of Medicine, University of Pennsylvania, Philadelphia, PA 19104, USA; mohaab@pennmedicine.upenn.edu; 2 Department of Microbiology and Immunology, Faculty of Pharmacy, Cairo University, Kasr El-Aini, Cairo 11562, Egypt

**Keywords:** LCMV, HIV, HCV, MHC restriction, tetramer, checkpoint blockade, immunotherapy, T cell exhaustion

## Abstract

Virology has played an essential role in deciphering many immunological phenomena, thus shaping our current understanding of the immune system. Animal models of viral infection and human viral infections were both important tools for immunological discoveries. This review discusses two immunological breakthroughs originally identified with the help of the lymphocytic choriomeningitis virus (LCMV) model; immunological restriction by major histocompatibility complex and immunotherapy using checkpoint blockade. In addition, we discuss related discoveries such as development of tetramers, viral escape mutation, and the phenomenon of T-cell exhaustion.

## 1. Introduction

The relationship between the disciplines of immunology and virology is a long intertwining one that started historically hundreds of years ago. Even before the establishment of either virology or immunology as a distinct scientific discipline, viruses provided a platform for demonstrating how the immune system works. For example, the principle of immunological memory that initiated the idea of vaccination was originally inspired by smallpox virus, and dates several centuries back to the tradition of inoculation or variolation by Asian cultures. It was based on the observation that individuals who survive smallpox disease once, become immune to the disease for the rest of their lives. In the late 18th century, Edward Jenner was the first to scientifically investigate vaccination and systematically vaccinate individuals with the less virulent cowpox virus to confer protection against the closely related smallpox, which is highly virulent and lethal [1]. A similar effort was performed by Louis Pasteur against another virus, rabies, almost a hundred years later. With better hypotheses about pathogens (the germ theory of disease) and human defense mechanisms, Pasteur made profound and valuable additions to Jenner’s vaccination scheme, by deliberately making the virus attenuated to be safe for administration as a vaccine [2]. The roads of virology and immunology often cross, that many attribute the birth of both the disciplines of modern immunology and modern virology at the end of the 19th century to the same scientist, Pasteur.

The viral kingdom with its rich diversity includes a plethora of viruses that target different organs in various host species, and possess a wide spectrum of viral-host interactions. This provided an ideal tool to study several immunological phenomena in mammals. The variations in hosts, targeted niches, and interactions enabled drawing many conclusions about immunological phenomena that are conserved across species and under different conditions [3,4,5,6]. Viruses represent the simplest class of mammalian pathogens compared to bacteria and eukaryotic parasites, with the majority of pathogenic mammalian viruses having a small number of proteins and simple genomic arrangement [7,8]. This limited number of genes and encoded proteins is a major advantage over other classes of pathogens as it facilitates dissecting immune responses against these few proteins, as well as identify interactions between viral proteins and host proteins. Additionally, with a limited arsenal of virulence factors compared to other classes of pathogens, it is less complicated to define associations between viral proteins and the pathology caused by infection.

**Figure 1:**
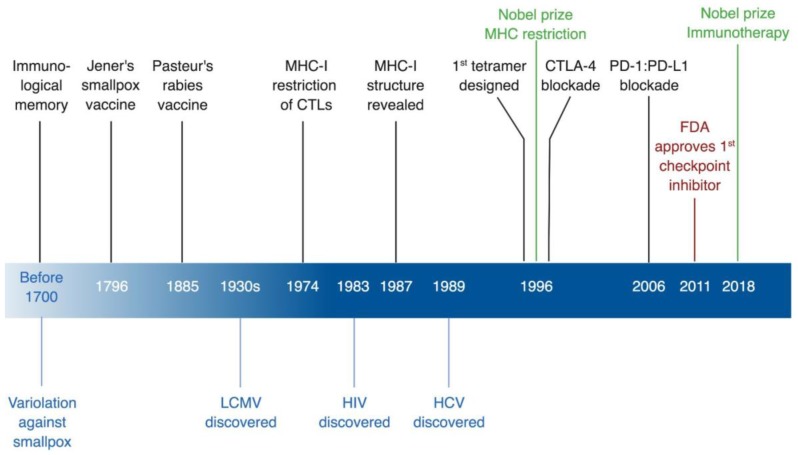
Timeline of immunological discoveries guided by viruses. In black, immunological discoveries, in green, related Nobel Prizes, in red, FDA approvals, and in blue virological discoveries. CTLs, cytotoxic T lymphocytes; CTLA-4, cytotoxic T lymphocyte antigen 4; FDA, US Food and Drug Administration; HCV, hepatitis C virus; HIV, human immunodeficiency virus; LCMV, lymphocytic choriomeningitis virus; MHC-I, MHC class I; PD-1, programmed cell death-1; PD-L1, programmed cell death ligand-1. * Created with BioRender.

There are numerous contributions of viral models and viral infections to immunological discoveries, and many of them were previously discussed by other reviews [9]. This review will focus on two milestones that revolutionized the field of immunology and had a great impact on its advancement. Specifically, the review will discuss the pivotal role of viral animal models in the discovery of immunological restriction by major histocompatibility complex (MHC) in mice [10,11], and the technical advance of developing tetramers based on this discovery [12]. In parallel, the review will discuss the impact of studying the human counterpart of MHC, the human leukocyte antigen (HLA), on the observations of escape mutation and protective HLA alleles in the context of human viral infections [4,5,13,14,15,16]. Additionally, the review will discuss the recent breakthrough in immunotherapy using checkpoint blockade [17,18,19,20], and the immunological phenomenon of T-cell exhaustion that served as the basis for this therapeutic strategy, a phenomenon that was also initially described in a virus mouse model [6,21,22] (Figure 1).

## 2. MHC Restriction

One of the most told success stories of viruses teaching immunology, is how the lymphocytic choriomeningitis virus (LCMV) mouse model enabled deciphering an important aspect of adaptive immunity, which is immunological restriction by the major histocompatibility complex (MHC). A main distinction between adaptive immune cells and innate immune cells is the high specificity of adaptive cells in recognizing specific foreign antigens. In the case of T lymphocytes this necessitates the processing of these antigens and presenting them to T cells as smaller peptides, known as epitopes. T cells recognize foreign antigens using T-cells receptors (TCRs). TCRs recognize foreign epitopes within the context of MHC proteins as a complex, the peptide-MHC complex (pMHC) [23,24,25,26].

LCMV was first discovered in the 1930s as the causative agent of lymphocytosis and meningeal inflammation in mice and humans [27,28]. LCMV is a positive single-stranded RNA (+SS RNA) virus with ~10.7 kb genome formed of 2 RNA segments [8,29]. LCMV proved to be an invaluable model for studying viral pathogenesis and immune responses for several reasons. First, the different strains of LCMV, and variations of both the route of infection and the age of mice at time of infection provided a wide spectrum of immune responses; ranging from tolerance at one end of the spectrum, to an efficient immune response in the setting of an acute resolving infection, and at the opposite end of the spectrum immune dysfunction causing chronic infection. Second, being a non-cytolytic virus, pathogenesis and tissue damage are almost exclusively due to immune responses, allowing accurate measurement of the cytotoxic activity of immune cells. Additionally, a major advantage of LCMV model is that most immunological findings from the model could be extended to human chronic diseases (reviewed in [8]).

The fashion by which T cells recognize epitopes was a complete mystery until the mid-1980s. On the other hand, between the early 1960s and early 1970s, extensive studies on B lymphocytes (at the time known as antibody-forming cell precursors) showed that surface antibodies existed on the surface of B lymphocytes, and it was suggested that these immunoglobulins were acting as the B-cell receptor (BCR) that binds their specific unprocessed antigen [30,31]. By 1973, it was shown that B cells can directly recognize antigens using multiple BCRs on the surface of the same B cell, all of these BCRs having the same specificity [32]. Nevertheless, it remained unknown whether T lymphocytes (thymus-derived lymphocytes) adopted a similar recognition system that directly recognizes antigens using immunoglobulin or immunoglobulin-like receptors, or whether T cells adopted a different recognition mechanism. The former theory was a popular assumption in the field in the early 1970s [33,34].

The recognition system of antigens by T cells started being deciphered by several groups in the mid-1970s [35,36,37,38,39], and a set of studies by Peter Doherty and Rolf Zinkernagel published between 1974 and 1975 clearly demonstrated a role of MHC in the ability of cytotoxic T lymphocytes (CTLs) to perform their function of lysing virus-infected cells [10,11,40,41,42]. These studies eventually earned them the Nobel Prize in physiology and medicine in 1996 “for their discoveries concerning the specificity of the cell mediated immune defense”.

**Figure 2:**
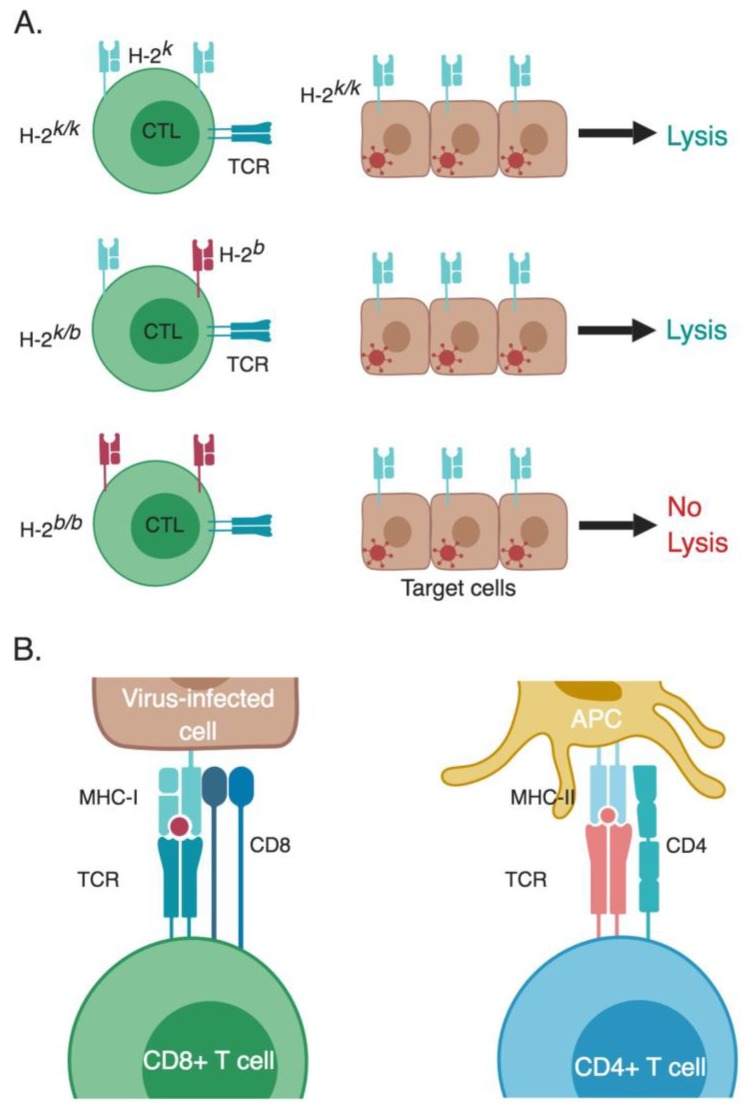
MHC restriction of T-cell responses. (**A**) Doherty and Zinkernagel experimental design for their Nobel Prize winning studies. They tested the ability of splenocytes from LCMV-infected mice with different H-2 backgrounds to lyse LCMV-infected mouse fibroblasts with an H-2^k/k^ background. Only CTLs that are H-2-compatible with the target cells were able to lyse them (in this specific experiment possessed at least one allele that is H-2k, either H-2^k/k^ or H-2^k/b^). Non-infected target cells co-cultured with CTLs having a matching MHC H-2 served as a negative control (not shown in the figure). (**B**) CD4+ and CD8+ T cells recognize their cognate epitopes that are presented within the context of MHC class II and I, respectively. These peptides are processed from foreign antigens and then presented to T cells. APC, antigen-presenting cell; CTL, cytotoxic T lymphocyte; MHC, major histocompatibility complex; TCR, T-cell receptor. * The different cells, receptors, ligands, and molecules are not drawn to scale. ** Created with BioRender.

Initially, MHC was recognized as a strain-specific protein expressed as distinct alleles on the cells of different mouse strains, causing rejection when organs or tissue are transferred from a different strain of mice [43]. However, the Oldstone laboratory published a study in 1973 that mice with different alleles of the MHC protein H-2 exhibited differential patterns of pathology in intra-cerebral (I.C.) infection by LCMV, indicating a link to LCMV pathogenesis [44]. In 1973, Doherty and Zinkernagel were originally exploring the role of CTLs in causing the lethal choriomeningitis following I.C. infection with LCMV. They showed that CTLs induced by LCMV infection are potent antiviral CTLs, since they caused destruction of LCMV infected target cells [45]. These experiments suggested that CTLs were causing the immunopathology and damage of the blood-brain barrier causing the acute brain edema characteristic of LCMV infection [46]. Together with the results from the Oldstone lab, Doherty and Zinkernagel designed a follow-up study that was initially intended to correlate the severity of the CTL-induced immunopathology of LCMV infection with the H-2 haplotypes, as they hypothesized that having different H-2 haplotypes would impact the level of CTL lytic activity. They tested the lytic activity using CTLs from spleens of LCMV–infected mice bearing different H-2 haplotypes, using LCMV-infected mouse fibroblasts (L929) as target cells. Only CTLs from some mouse groups were able to lyse infected target cells, although mice from all groups had developed the same lethal LCMV disease between days 7–12. Initially, they thought something went wrong with the experiments, so they revised all of the data concerning the sources of mice and cells, including the L929 cells that were used as target cells. They observed that all of the target L929 cells were from the CBA/H mouse strain, which has an H-2^k/k^ allele. All CTLs that were able to successfully lyse the H-2^k/k^ LCMV-infected target cells possessed at least one allele that is H-2k (either H-2^k/k^ or H-2^k/b^) [47] (Figure 2A). This suggested that there might be a correlation between having matching MHC H-2 on both CTLs and target cells in order for CTLs to be functional and affect lysis of target cells. To confirm the requirement of having matching MHC alleles between target cells and CTLs for efficient cytotoxicity, they tested mice from other H-2 backgrounds, and confirmed that CTLs were only able to lyse target cells that are H-2-compatible [10,41]. As a control, they co-cultured non-infected target cells with CTLs having a matching MHC H-2 to confirm that the killing was specific to LCMV-infected cells.

Previous studies from Benacerraf lab, as well as Kindred and Shreffler labs had already shown a similar role for MHC compatibility for successful B-cell responses, where they showed that helper activity provided by CD4+ T cells for B cells were only possible between histocompatible T and B cells [48,49,50]. Other studies also confirmed a role for MHC in CTL activity [35,36,37,38,39].

These studies by Zinkernagel and Doherty capitalized on previous findings through decades of virological studies that defined various aspects of LCMV infection and the characteristics of various LCMV strains, as well as the impact of the different routes of infection on the kinetics, pathology, and outcome of infection [51,52,53,54,55].

By the late 1970s great strides have been made in the field of T cell immunology, and many discoveries followed that completed the missing pieces of the puzzle. The structures of MHC class I and II were revealed in 1987 [56] and 1993 [57], respectively. The TCR was identified in 1982/1983 [58,59,60], and by 1984 the genes encoding the β chain of the TCR in both mice and humans were cloned [61,62], followed by the gene encoding the α chain, which culminated in the elucidation of the TCR structure [63]. This was followed by the discovery of antigen processing pathways that elucidated the different steps for processing foreign antigens to produce epitopes that could be presented to either CD8+ and CD4+ T cells within the context of MHC class I and class II, respectively [23,24]. This led to an understanding of how naïve T cells become primed and activated to differentiate into effector T cells (Teff) able to combat pathogens and foreign antigens (Figure 2B).

## 3. Tetramer Development and Related Discoveries

Many findings followed the discovery of MHC restriction of T-cell responses and the TCR structure, and those findings elucidated the highly specific interaction between the pMHC and TCR. A major technical innovation was the development of tetramers as a major tool that advanced immunological research [12]. Immunological studies that followed the development of tetramer staining unraveled the deep impact of this highly specific pMHC-TCR interaction on both sides of the relationship; the virus and the host. Thorough examination showed that particular mutations at specific sites in the viral genome are driven by immunological pressure from the host T cells targeting specific epitopes [4,5]. On the other hand, specific MHC alleles have been shown to confer superior protective capacity for individuals who carry them against specific viral infections [13,14,15,16], and genome wide association studies (GWAS) identify HLA genes as one of the top genes correlating with disease development in many infection contexts, including HIV and hepatitis B virus (HBV) (reviewed in [64]). Finally, tetramer development enabled elucidation of the biology of different phenotypes of the same epitope-specific T cells in the context of different conditions of infection (e.g. acute versus chronic) [21,22].

### 3.1. Tetramers

**Figure 3:**
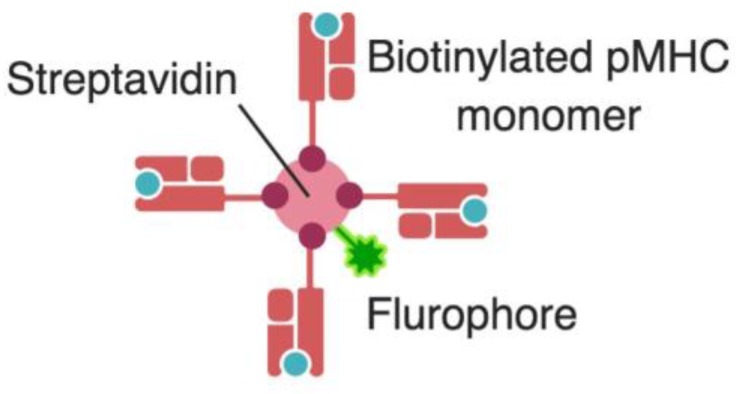
Tetramer design. A biotinylated MHC molecule is loaded with an epitope of known specificity that is optimally presented by this specific MHC allele. Four of these biotinylated pMHC molecules are tetramerized by binding them together with a streptavidin molecule bearing a fluorophore that could then be detected by flow cytometry. pMHC, peptide-MHC complex. * Created with BioRender.

Following the set of findings that culminated in the elucidation of pMHC:TCR interaction, and capitalizing on the advances in flow cytometry, an important tool for immunologists was developed, tetramers. With the knowledge that specific epitopes are optimally presented by specific MHC alleles, the principle of tetramer design is to load a known epitope on an MHC molecule of the best fitting allele that optimally presents this epitope, thus maximizing the sensitivity of the binding and detection of specific T cells that recognize this epitope. Four biotinylated pMHC molecules are then tetramerized by binding them together with a streptavidin molecule bearing a fluorophore (Figure 3).

Tetramer design and usage for tracking epitope-specific T cells was first reported by the group of Mark Davis in 1996 [12]. Staining with tetramers allows the detection of epitope-specific T cells by flow cytometry, and recently mass cytometry using cytometry by time-of-flight (CyTOF) [65]. Tetramers became one of the immunologist’s best friends, as it allowed the tracking of pathogen-specific responses, especially of well-defined viral epitopes versus immune profiling in the pre-tetramer era that used to profile total T-cell responses. Tracking total T-cell responses had many limitations, as it would include responses that are non-specific to the pathogen in question, and it does not enable distinguishing the kinetics of individual immune responses to each epitope, especially for epitopes originating from different viral proteins. Indeed, using tetramers it became obvious that T cell responses to epitopes from different viral proteins have different kinetics [21]. These different kinetics are expected, since the expression kinetics of the different proteins are different at the different stages of viral life cycle [29]. Thus, tetramers enabled the tracking of epitope-specific T cells and dissecting immune responses with high resolution by distinguishing immune responses to different epitope specificities belonging to the same pathogen [21]. Currently, up to 10 monomers could be linked together forming a dextramer. Dextramers enable better detection of epitope specific T cells with high sensitivity, especially for pMHC:TCR with low affinity [66].

### 3.2. Escape Mutation

With a low fidelity polymerase that lacks a proofreading function, RNA viruses have an exceptionally high incidence of mutations. As they co-evolved with different hosts, the fittest sequences that kept arising throughout their replication cycles were continuously selected for [7]. One example of an RNA virus with a high mutation rate is hepatitis C virus (HCV), with an RNA-polymerase that has a mutation rate of ~2/1000 bases per year [67]. HCV replicates rapidly with an average half-life of ~3 h, giving rise to 10^12^ copies in a single host in one day, which means that thousands of new sequences arise daily in an individual [68]. Notably, a higher mutation rate was observed in the HCV epitope-regions targeted by virus-specific T-cells versus other regions outside the epitope regions, where the rate of mutation was 13-folds higher in the epitope regions [69]. An HIV study also showed that two-thirds of all non-synonymous mutations were due to CD8+ T-cell selective pressure [4]. Similar scenarios were shown for SIV and in other HIV studies [70,71].

HIV and HCV are both +SS RNA viruses with a genome of ~9 Kb and ~10 Kb, respectively [72,73]. Whereas HIV establishes persistent infection in all individuals who become infected, 25–30% of HCV-infected patients spontaneously clear the virus [73,74]. Both HIV and HCV were extensively studied virologically which facilitated the explanation of immunological phenomena associated with their corresponding infections.

Viral escape was first suggested in the LCMV model, even before tetramer development [3]. Nevertheless, with tetramer development, it became feasible to follow T cell responses to a specific viral epitope, and at the same time sequence the targeted epitope longitudinally at different time points corresponding to different stages of the infection and disease. It became obvious that one of the mutational hotspots for with pressure from the adaptive arm of the immune response is T-cell epitopes [4,69]. This strategy is adopted by the virus to “escape” from immune responses that could achieve viral clearance, and is most notable in immunodominant epitopes that are heavily targeted by the immune system. By undergoing mutations in the amino acids within the epitope sequence, especially the anchor residues that are most important for binding to the MHC protein, the virus evades optimal binding by the MHC molecule, and thus cannot be optimally presented by antigen presenting cells (APCs) or recognized by the corresponding specific T cells [4,75,76,77]. Nevertheless, non-synonymous amino acid mutations in the flanking regions of the epitope could also lead to viral escape by disrupting epitope processing, thus compromising optimal antigen recognition by the specific T cells [71,78].

This imprinting of the immune system on viral sequences at specific epitope locations could be observed in individuals with specific HLA alleles. This is mainly due to the ability of these HLA alleles to present these epitopes to T cells more efficiently, thus exerting an immune pressure driving the mutation. For example, within the non-structural (NS) proteins region of HCV encoding proteins NS2 to NS5A/B, epitopes NS3-1073 and NS3-1406 mainly undergo mutations in HLA-A2 individuals, whereas the NS3-1436 epitope undergoes mutation in HLA-A1 individuals [75,76,77,79,80,81]. For HIV, selection of a mutation altering an amino acid in the flanking region of the group-specific antigen (Gag) kk9 epitope usually occurs in HLA-A3 individuals [71]. This HLA-specific influence on viral escape was clearly demonstrated by closely monitoring the mutated epitope sequence upon transmission to another individual lacking the best-fitting HLA allele (that was present in the original patient), where the epitope reverts to the original sequence that ensures optimal replication of the virus [4,5].

### 3.3. Protective HLA Alleles

Another aspect of the interplay between T cells and viruses that was revealed as a consequence of MHC restriction discovery and development of tetramers, was the fact that some individuals possess specific HLA alleles that could be more protective against specific viral infections. For example, HLA-B*27 and HLA-B*57 were suggested to confer higher degree of protection against progression of disease in HIV [4,13,14,82,83]. Disease progression in HIV is measured by two factors; the decrease CD4 T cells count and the viral load. Long-term non-progressors (LTNP), are patients who have been infected with HIV for more than 10 years, yet their CD4 T cells count remains almost normal and HIV viral load is very low, thus they do not progress to AIDS [14]. It has been reported that possessing either an HLA-B*27 or HLA-B*57 was associated with LTNP [13,14,82,83]. For HLA-B*27 this protection was associated with a strong immune response against the highly conserved and immunodominant epitope p24 in the Gag protein region [82]. HLA-B*57 protective capacity was associated with targeting multiple epitopes in the Gag and polymerase (pol) regions of HIV sequence [4,13,83].

Interestingly, the same HLA alleles protected against other types of infection, where individuals possessing either have a higher chance of clearing HCV spontaneously during acute phase of infection [15,16,84,85]. It is suggested that these HLA alleles possess superior protective capacities, since the epitopes they target are essential for the viral life cycle and have a high fitness cost if they get mutated, thus T-cell responses restricted by these HLA alleles have a higher ability to control the infection. For example, HLA-B*27 targets the NS5B-2841 epitope that is highly conserved in HCV genotype 1, and the virus suffers a high fitness cost to escape this specific response [85]. Thus, individuals bearing the HLA-B*27 allele have a higher chance of clearing HCV spontaneously [15,84,85].

HLA-B*27 seems to be a “super” allele, since it has also been associated with a better outcome of disease in infections with influenza A virus, Epstein-Barr virus, and herpes simplex virus. Nevertheless, everything comes at a price, since HLA-B*27 has a strong association with inflammatory diseases, and HLA-B*27+ individuals have a higher probability of developing ankylosing spondylitis, Reiter’s syndrome, as well as other inflammatory diseases [14].

Epitopes mentioned in the previous sub-section, such as HCV-NS3-1073 and HIV-Gag-kk9, have a low genetic barrier (where 1 nucleotide mutation causes amino acid changes), as well as a low fitness cost to the virus that suffers non-significant effect on its replication [4,76]. In contrast, epitopes targeted by protective HLA alleles often require a complex array of mutations to escape, and it comes at a high fitness cost since viral replication is highly compromised [13,14,82,83].

### 3.4. LCMV; Armstrong versus Clone-13

Another important addition to immunology achieved using tetramers was the accurate phenotyping of virus-specific T cells in various immunological settings. Another major contribution of the LCMV field, was the isolation of a mutated strain of the original LCMV Armstrong (LCMV-Arm) with a very similar sequence that is able to establish chronic infection in adult mice with an intact immune system, as opposed to LCMV-Arm that causes acute infection. Rafi Ahmed, at that time a postdoctoral fellow in the Oldstone lab, reported the isolation of several mutant strains of LCMV-Arm. Clone number 13 isolated from the spleen (ever since famously known as the “Clone 13” strain), had different infection kinetics than LCMV-Arm, as well as a unique pathology [86]. LCMV-Arm has an acute onset of infection causing severe inflammation in the meninges, but the infection spontaneously clears within the first 10 days post infection. On the other hand, LCMV clone 13 (LCMV-cl13) infection establishes viremia for up to 90 days, and establishes a life-long reservoir in the brain and kidney [87]. A great advantage of LCMV-cl13 is that this strain has nearly the same genomic sequence as the LCMV-Arm, where the two strains only differ by 3 amino acids [88,89,90]. Importantly, none of these amino acids map within T cell epitopes, thus all epitopes could be tracked side-by-side in the two infection settings, acute and chronic [21,22,87,91]. This enabled accurate description of the status of virus-specific T cells at different stages of differentiation in the setting chronic infection contrasted to acute infection. This was achieved by tracking T cells specific to the same epitopes using tetramers in the setting of an acute resolving LCMV-Arm infection versus a chronic LCMV-cl13 persistent infection. This eventually enabled the accurate definition of the phenotype, and essential transcriptional circuits for T cells to differentiate into dysfunctional exhausted T cells during chronic infection, in contrast to functional effector cells during the response to acute infection, that then give rise to long-lived memory T cells following clearance of the infection [21,22,91,92,93,94]. Exhausted T cells will be discussed in detail in the next section.

Another advantage of the LCMV-cl13 system is that by inducing CD4 T cell depletion at the initiation of infection, LCMV-cl13 would establish a chronic viremia for the life of the mouse [87]. This is different from normal LCMV-cl13 infection that establishes viremia for up to 90 days, then the virus becomes undetectable in the blood and most organs, except for the brain and kidney where it establishes a life-long reservoir [87]. LCMV-cl13 infection with CD4 T cell depletion is a better mirror of the kinetics of many human chronic viral infections where there is an initial stage of rapid viral replication resulting in a high viral load, followed by a balance between the immune response and viral replication that results in a plateau of viral load at a lower level than the initial peak that persists for the life of the host [87,95].

Collectively, the findings discussed in this section demonstrate how thousands of years of virus-mammal coevolution has left clear marks on both. The mark imprinted by the host on the virus is mainly due to the adaptive arm of the immune response imposing selective pressure on specific locations within the viral sequence that represent targeted epitopes. The virus escapes from the immune pressure through a low-fidelity polymerase that inflicts a high error rate and permits selection of mutants that are not efficiently recognized by the immune system. As with all mutations, the fitness cost seems to be a very decisive point on whether the produced mutant genome will become fixed in the niche or whether it is replication defective and thus rapidly disappears. These findings were made possible following identification of the role of MHC/HLA in immunological restriction of T-cell responses using the well-established viral model of LCMV, and development of tetramers. Moreover, within the context of the LCMV model and using tetramers, it became possible to track the differentiation of T cells targeting the same specific epitope in both settings of an acute infection by the Armstrong strain and in a chronic infection by the clone-13 strain.

## 4. Immunotherapy and T-Cell Exhaustion

Immunotherapy is the most recent immunological breakthrough that revolutionized therapeutic strategies against cancer and other chronic diseases. Novel immunotherapies include genetically engineered T cells known as chimeric antigen receptor T cells (CAR T cells) [96], viruses that target and lyse tumor cells (Oncolytic viruses) [97], and vaccines targeting newly generated antigens due to tumor mutations (neoantigens) [98,99]. The first two approaches received FDA approval between 2015 and 2017, and the third strategy is in phase III clinical trials [100]. Nevertheless, the strategy that sparked the immunotherapy revolution since 2006 by showing highly promising results during clinical trials was checkpoint blockade. Clinically, since 2011 checkpoint inhibitors achieved great success in patients resistant to other traditional therapeutic strategies such as chemotherapy and radiation, thus becoming the standard of care for many cancer types [19]. So far, single and combined checkpoint blockade have been approved in 11 tumor types including melanoma, non-small cell lung carcinoma (NSCLC), and head-and-neck cancer [101].

Checkpoint inhibitors are monoclonal antibodies that block interaction between inhibitory receptors (IRs) and their ligands, such as antibodies blocking the PD-1:PD-L1 pathway (Programmed death-1:Programmed death-ligand1) and antibodies blocking CTLA-4 (Cytotoxic T cell ligand-4). These IRs are expressed on the surface of dysfunctional T cells during chronic viral infection and in the context of many tumors. Currently, there are several checkpoint inhibitors available on the market, such as the anti-CTLA-4, ipilimumab, anti-PD-1, nivolumab and pembrolizumab, as well as anti-PD-L1, atezolizumab, durvalumab, and avelumab. Checkpoint blockade provided new hope for patients with cancer refractory to classical strategies of treatment, since monotherapy with PD-1:PD-L1 inhibitors or in combination with anti-CTLA-4 had high response rates ranging 50–90% of patients with Hodgkin’s lymphoma and Merkel cell carcinoma, and ~40% for melanoma [101,102,103]. Combining PD-1:PD-L1 blockade with other checkpoint inhibitors has promising synergistic effects, especially for IRs with distinct mechanisms of action [104]. In 2018, this immunotherapy revolution culminated in Dr. James P. Allison and Dr. Tasuku Honjo being awarded the Nobel Prize in medicine “for their discoveries in the field of cancer immunotherapy by blocking negative immune regulation”. All of this had a great push with a breakthrough study in the LCMV mouse model [18]. In addition, the basic immunology of dysfunctional T lymphocytes that were shown to mediate the therapeutic effects of checkpoint blockade was also defined in the context of the LCMV mouse model.

**Figure 4:**
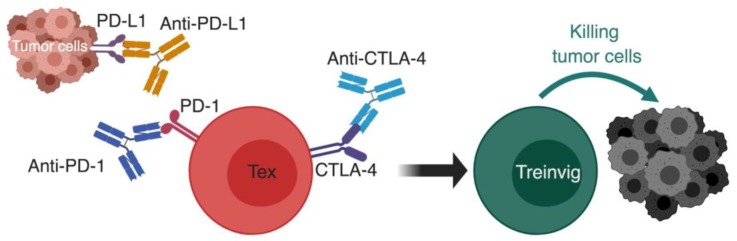
Using checkpoint blockade to reinvigorate exhausted T cells. The basic principal of immunotherapy by checkpoint blockade; monoclonal antibodies against inhibitory receptors such as PD-1:PD-L1 pathway and CTLA-4 are used to block the inhibitory signals on exhausted T cells, which causes their reinvigoration and restoration of effector functions that causes successful killing of tumor cells. CTLA-4, cytotoxic T lymphocyte antigen 4; PD-1, programmed cell death-1; PD-L1, programmed cell death ligand-1; Tex, exhausted T cell; Treinvig, reinvigorated T cells. *The different cells, receptors, ligands, and molecules are not drawn to scale. ** Created with BioRender.

Originally, Dr. Allison’s idea was to block the interaction between inhibitory receptors and their ligands, thus preventing the negative regulation of immune responses. Following the discovery of CTLA-4 in 1987 and Allison demonstrating its inhibitory effect on T cell functions [105,106], he sought to interrupt those inhibitory signals using a blocking antibody against the inhibitory receptor CTLA-4 in a mouse model of cancer. Treatment with anti-CTLA4 enhanced the rejection of colon carcinoma and protected the animals against subsequent challenges, as well as reducing the growth of murine fibrosarcoma [17]. This CTLA-4 blockade study was a proof-of-principle that checkpoint blockade could be a strategy for controlling chronic disease (Figure 4). Nevertheless, a breakthrough came from a study in the LCMV model by Barber et al. from Rafi Ahmed’s group in 2006. This study demonstrated that blocking the interaction between another inhibitory receptor, PD-1, and its ligand PD-L1 could be very highly effective in controlling chronic infection by LCMV-cl13 [87] (Figure 4). An additional mechanistic insight from this study was elucidating that viral control was associated with reversal of dysfunctionality of virus-specific T cells, which normally suffer diminished effector functions during chronic LCMV-cl13 infection [21,107]. Thus, the discovery that blocking the PD-1:PD-L1 pathway could reverse dysfunction of CD8 T cells during chronic disease was first made in a viral infection model [18]. This study demonstrated clearly that the control of LCMV-cl13 infection (and most probably the control of tumor growth in the Allison lab study) was a result of reinvigoration of dysfunctional virus-specific T cells, and that this reinvigoration was directly correlated to blocking the PD-1:PD-L1 pathway, and consequently preventing the inhibitory signals propagated through PD-1 that induce the dysfunctional state of T cells.

The study by Barber et al. was founded on previous studies that described the immunological phenomenon known as T cell exhaustion, that was further explored by later studies as well [21,22,91,92,93,95,108]. The majority of research performed to define T cell exhaustion was also primarily conducted in the LCMV mouse model. T-cell exhaustion represents a state of T-cell dysfunction and a differentiation pathway of T cells that occurs upon persistent antigen stimulation [6]. Exhausted T cells (Tex) are characterized by their dysfunction compared to functional effector T cells (Teff) or memory T cells (Tmem) that are generated during acute infection and after its resolution, respectively. Tex display gradual and hierarchical loss of production of antiviral/antitumor cytokines, as well as diminished cytotoxic function [21,107]. This represents a main reason for the failure of the immune system to clear chronic viruses and eradicate tumor cells. In addition to their dysfunction, Tex are characterized by a unique phenotype, as well as a unique transcriptional and epigenetic profile [22,93,94,109]. One phenotypic hallmark of Tex is the sustained elevated expression of a group of markers that collectively became known as inhibitory receptors (IRs). The most well-characterized inhibitory receptor is PD-1 which seems to be ubiquitously expressed on Tex in different chronic viral infections and cancers [110]. The main effect of PD-1 upon binding to its ligands is sending inhibitory signals that counteract the effects of stimulatory and co-stimulatory signals that follow T cell activation [110,111,112]. These inhibitory signals from the PD-1 pathway eventually lead to diminished T cell functions. PD-1 exerts its inhibitory action following binding to its ligands PD-L1 and PD-L2 that are highly expressed on tumor cells and virus-infected cells [113]. Some IRs are also transiently expressed on Teff during the contraction phase of the immune response following resolution of an acute infection. From an evolutionary perspective, this suggests that IRs originally evolved to act as checkpoints or “brakes” for the immune system to prevent over-activation of the immune system and to limit potential immunopathology that could result from an exaggerated immune response [114].

**Figure 5:**
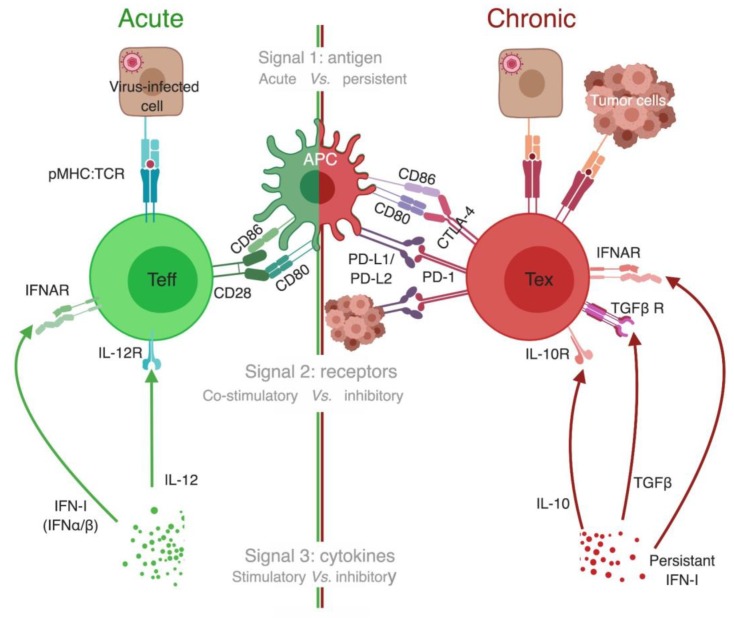
Signals for development of T cell exhaustion. Three signals’ model has been previously proposed to explain optimal development of Teff (left half of the figure, green color code). We propose a similar three signals’ model for exhausted T cell (Tex) development (right half, red color code). Signal 1 in acute infections is provided by a brief encounter with the antigen, whereas in chronic infection and cancer there is persistent antigen stimulation. Antigen binding alone is not enough to activate naïve T cells and could even drive them to become anergic [117,118]. Signal 2 for Teff is manifested by upregulation of the expression of co-stimulatory receptors on T cells (such as CD28) that bind ligands on APCs (such as CD80 and CD86), which primes naïve T cells to enter the effector program. In contrast, Tex receive an opposite signal 2 from inhibitory receptors (IRs) that are highly expressed on their surface. IRs either competitively bind to co-stimulatory ligands, as with CTLA-4 that binds CD80/CD86 preventing them from binding to CD28, or bind to unique ligands as with PD-1 binding to PD-L1/PD-L2. In either case, IRs’ ligation to their corresponding ligands initiates a cascade of inhibitory signals that gradually diminishes functionality of Tex [110,114]. Signal 3 for optimal activation of T cells takes the form of brief exposure to stimulatory and inflammatory cytokines, such as interleukin-12 (IL-12) and type I interferon (IFN-I) [115,116]. In chronic infection, there is a persistent elevated level of IFN-I in many contexts, and blocking IFN-I signaling enhances viral control [119,120]. Additionally, cytokines known to have an immunosuppressive effect are elevated in chronic infection, such as IL-10 and transforming growth factor (TGFβ) [121,122]. APC, antigen-presenting cell; CTLA-4, cytotoxic T lymphocyte antigen 4; IFNAR, type I interferon receptor; IFN-I, type I interferon; IL, interleukin; IL-10R/ IL-12R, receptor for interleukin 10 and 12, respectively; PD-1, programmed cell death-1; PD-L1/ PD-L2, programmed cell death ligand-1/2; pMHC:TCR, peptide-MHC:T-cell receptor complex; Teff, effector T cells; Tex, exhausted T cell; TGFβ, transforming growth factor beta; TGFβ R, TGFβ receptor. *The different cells, receptors, ligands, and molecules are not drawn to scale. **Created with BioRender.

As discussed in the previous section, tetramers allowed direct comparison of T cells targeting a specific epitope in both acute and chronic LCMV infection settings, and track their differentiation. Similar to the 3 signals needed for priming and activation of naïve T cells to generate Teff [115,116], 3 signals seem to be involved in the development of Tex (Figure 5).

Although the phenomenon of T cell exhaustion was mainly characterized in the LCMV mouse model, it became clear that T cell exhaustion is a hallmark of chronic viral infections in general. Tex were described for various viral models (e.g., HIV infected humanized mice, and SIV infected rhesus macaques) [123,124], as well as human viral infections such as HIV [125], HBV [126], and HCV [127]. These findings were extended to the cancer field, where tumor-infiltrating lymphocytes (TILs) displayed the main features of Tex in tumor mouse models, such as chronic myeloid leukemia model [128], and liver cancer model [129], as well as numerous human cancers, including melanoma [130], non-small cell carcinoma [131], Hodgkins lymphoma [132], ovarian cancer [133], and chronic lymphocytic leukemia [134].

Taking into consideration the success rates of anti-PD-1:PD-L1 therapy, there are currently ~481 active clinical trials testing anti-PD-1:PD-L1 monotherapy, and more than 1132 in combination with other immunotherapeutic strategies. During the past year, the US Food and Drug Administration (FDA) has granted approval to 13 additional indications for anti-PD1:PD-L1 therapies, including a new anti-PD1 agent [20]. In addition, the FDA is granting accelerated approvals to other immunotherapies, and has approved two CAR T therapies in the past few months [19]. Thus, immunotherapy still holds great potential, and clinical trials are expanding not only for checkpoint inhibitors, but also for other immunotherapeutic strategies. In the period between 2017 to 2018, there was a 34% increase in immunotherapeutic agents tested in clinical trials (currently 1287 agents), but the increase was more pronounced for pre-clinical agents with the number nearly doubled (97% increase, currently 2107 agents) [19]. This reflects that this immunotherapy revolution motivated a great surge in basic research in the field of immuno-oncology. Well-established and thoroughly characterized viral models such as the LCMV mouse model have a great potential and hold a promise for more discoveries enriching the immunotherapy landscape. The LCMV model still represents a cornerstone in current research towards a deeper understanding of the transcriptional and epigenetic mechanisms underlying failure of the immune system in face of chronic diseases, and discovering novel pathways to achieve reinvigoration and durable recovery of an effective and functional immune response.

## 5. Conclusions

Well-defined viral models in laboratory animals such as the LCMV mouse model and viral infections in humans, such as HIV and HCV represented an outstanding tool for the discovery of many immunological phenomena that drove the immunology field forward. Pivotal immunological discoveries such as MHC restriction of cellular adaptive immunity, defining T-cell exhaustion, and checkpoint blockade would not have been possible without thorough knowledge of viruses and viral models, and the ability to manipulate these models. Studying human viral infections confirmed several immunological discoveries, including viral escape mutation and protective MHC alleles. Thorough understanding of viruses and using novel technical advances to manipulate viral models is key for deeper understanding of many immunological phenomena, and translating these findings into therapeutically beneficial strategies.
